# Artificial Intelligence-Based Left Ventricular Ejection Fraction by Medical Students for Mortality and Readmission Prediction

**DOI:** 10.3390/diagnostics14070767

**Published:** 2024-04-04

**Authors:** Ziv Dadon, Moshe Rav Acha, Amir Orlev, Shemy Carasso, Michael Glikson, Shmuel Gottlieb, Evan Avraham Alpert

**Affiliations:** 1 Jesselson Integrated Heart Center, Eisenberg R&D Authority, Shaare Zedek Medical Center, Jerusalem 9103102, Israel; 2 Faculty of Medicine, Hebrew University of Jerusalem, Jerusalem 9112102, Israel; 3 Azrieli Faculty of Medicine, Bar-Ilan University, Safed 1311502, Israel; 4 Sackler Faculty of Medicine, Tel Aviv University, Tel Aviv 6997801, Israel; 5 Department of Emergency Medicine, Hadassah Medical Center—Ein Kerem, Jerusalem 9112001, Israel

**Keywords:** AI (Artificial Intelligence), echocardiography, point-of-care testing, students, medical, ventricular function, left

## Abstract

Introduction: Point-of-care ultrasound has become a universal practice, employed by physicians across various disciplines, contributing to diagnostic processes and decision-making. Aim: To assess the association of reduced (<50%) left-ventricular ejection fraction (LVEF) based on prospective point-of-care ultrasound operated by medical students using an artificial intelligence (AI) tool and 1-year primary composite outcome, including mortality and readmission for cardiovascular-related causes. Methods: Eight trained medical students used a hand-held ultrasound device (HUD) equipped with an AI-based tool for automatic evaluation of the LVEF of non-selected patients hospitalized in a cardiology department from March 2019 through March 2020. Results: The study included 82 patients (72 males aged 58.5 ± 16.8 years), of whom 34 (41.5%) were diagnosed with AI-based reduced LVEF. The rates of the composite outcome were higher among patients with reduced systolic function compared to those with preserved LVEF (41.2% vs. 16.7%, *p* = 0.014). Adjusting for pertinent variables, reduced LVEF independently predicted the composite outcome (HR 2.717, 95% CI 1.083–6.817, *p* = 0.033). As compared to those with LVEF ≥ 50%, patients with reduced LVEF had a longer length of stay and higher rates of the secondary composite outcome, including in-hospital death, advanced ventilatory support, shock, and acute decompensated heart failure. Conclusions: AI-based assessment of reduced systolic function in the hands of medical students, independently predicted 1-year mortality and cardiovascular-related readmission and was associated with unfavorable in-hospital outcomes. AI utilization by novice users may be an important tool for risk stratification for hospitalized patients.

## 1. Introduction

Point-of-care ultrasound (POCUS) is finding its niche in all fields of clinical medicine including among advanced care providers and nurses [1]. Certain academic societies were early adopters of POCUS. For example, two decades ago the American College of Emergency Physicians initially recommended six clinical ultrasound applications which have now expanded to 12 core applications ranging from cardiac, trauma, and pregnancy, to nerve blocks [2]. Other societies such as critical care medicine and anesthesia focused on hemodynamic assessment and procedural guidance, e.g., venous access and nerve blocks [3,4,5,6]. Most internal medicine residency programs in North America have incorporated POCUS into their curriculum, some have even started fellowships [7]. Also, family medicine is embarking on a project to certify educators in POCUS so that they can develop courses to teach fellow clinicians [8].

Meanwhile, the applications in all specialties are also expanding, particularly in the field of cardiac POCUS. This has included POCUS assessment by general emergency physicians in the setting of cardiac arrest, identifying those with cardiac motion but undetectable pulses. Such patients were found to have a higher percentage of return of spontaneous circulation and survival to hospital discharge [9,10]. The advantage of POCUS was also shown in assessing undifferentiated shock. While POCUS can be used to evaluate causes of shock including tension pneumothorax or ruptured abdominal aortic aneurysm, cardiac POCUS plays an important diagnostic role in identifying the presence of tamponade, myocardial infarction, or pulmonary embolus [11,12,13]. In the field of anesthesiology, cardiac POCUS is now being incorporated into the pre-operative assessment of patients, particularly including the evaluation of left ventricular (LV) systolic dysfunction and aortic stenosis [14,15].

In terms of estimation of LV ejection fraction (LVEF) by non-cardiologists including physicians as well as nurses, most of the literature focuses on their ability to categorize the LVEF into different functional groups, e.g., normal, moderately reduced, or grossly reduced, as opposed to committing to a specific value [16]. A consensus statement by the American Society of Echocardiography (ASE) and the American College of Emergency Physicians (ACEP) suggested that focused cardiac ultrasound in the hands of emergency physicians should assess the global cardiac systolic function categorizing the LVEF into the three mentioned groups [17]. Accordingly, Unlüer and colleagues showed that trained emergency physicians using POCUS could accurately visually identify the LVEF as either normal or low [18]. Also, internists in a study by Johnson et al. could accurately identify patients with a normal versus low LVEF [19]. Similarly, Kirkpatrick et al. showed that registered nurses in an outpatient diabetes clinic following a 4 h training could accurately identify the patients with reduced LV systolic function using a HUD [20]. Similar findings were shown regarding medical students when assessing the LVEF, affirming that by using a new visual approach for evaluating cardiac function using template matching they can accurately estimate LVEF [21]. However, this may not always hold true for novice users. One study on general practitioners using a HUD showed poor agreement with experts (Cohen’s kappa coefficient of 0.22; sensitivity, 47.4%; specificity, 81.0%) in identifying echocardiogram examinations with a reduced LVEF [22].

With the recent advances in ultrasound technology and image acquisition, several hand-held ultrasound devices (HUD) are commercially available, contributing to a further expansion of POCUS integration into many daily clinical practices [23]. For example, these miniaturized devices played an important role in the recent COVID-19 pandemic in terms of predicting patients having unfavorable outcomes [24]. One of their advantages that was incredibly valuable during the pandemic is their small surface area which allowed them to be easily decontaminated [25].

In parallel to POCUS, artificial intelligence (AI) is taking off exponentially and permeating all fields of medicine including improving diagnostic accuracy and image interpretation. Even though it may involve significant challenges, including implementation issues, accountability, and fairness, AI for medical purposes has made significant progress toward large-scale deployment, especially through medical image analysis and high-quality studies. Nonetheless, in the medical field AI remains in its early phases of validation [26]. The fusion of high-end AI capabilities with precision medicine holds the potential for revolutionized health care, incorporating sophisticated computation and inference to generate insights that enable the system to reason and learn, and empowers clinician decision-making through augmented intelligence [27]. This is particularly relevant to the field of cardiology where it has been shown to improve electrocardiogram interpretation [28], predict heart failure [29], and enhance echocardiogram interpretation [30].

In a recent prospective study, we showed that medical students with basic image acquisition skills who used a HUD with an automated proprietary AI program to assess LVEF, were able to achieve a degree of accuracy that was similar to that of board-certified cardiologists [31]. This present study is a continuation of this project with the objective of determining the ability of the HUD AI-based tool in the hands of novice users to predict outcomes of patients admitted to the cardiology department.

## 2. Materials and Methods

### 2.1. Study Design and Setting

This is a prospective observational study of real-time focused cardiac ultrasound using a HUD on non-selected patients admitted to the cardiology department. The images were obtained by medical students using an AI-based tool for the assessment of LV systolic function. The study was approved by the hospital’s Institutional Review Board (IRB number 001024-SZMC).

The study’s primary endpoint is the adjusted association between AI-based reduced LV systolic function (LVEF < 50%) and the primary composite outcome of 1-year mortality and readmissions for cardiovascular-related causes.

Secondary endpoints included an association between AI-based reduced LV systolic function and in-hospital secondary composite outcome including in-hospital death, advanced ventilatory support (high-flow nasal cannula, non-invasive positive airway pressure support, and invasive ventilation), shock (requiring intravenous pressors/inotropes support) and acute decompensated heart failure (ADHF; requiring intravenous diuretics). The following variables were also included in the secondary endpoints: individual parameters of the secondary composite outcome, acute kidney injury, LV/left atrial appendage thrombus, electrical cardioversion, any ventilatory support (nasal cannula or advanced ventilatory support), hospital length of stay (LOS) and cardiovascular implantable electronic device insertion.

Study participants included patients ≥ 18 years old admitted to the cardiology department at a single tertiary care medical center from March 2019 through March 2020. Participants were recruited within their first 48 h of hospitalization.

The study involved medical students trained to utilize a HUD equipped with an AI-based tool that provides automated calculation of the LVEF. The students, all working as medical assistants in the cardiology department, were assigned to an educational program comprised of face-to-face lectures and practical training sessions. The 6 h of lectures addressed ultrasound principles and different cardiac ultrasound clinical applications, including a presentation of different cardiac ultrasound clips covering the full spectrum of LVEF calculation using different views. During the practical training sessions, each trainee was required to perform four echocardiography clip acquisitions under supervision. All acquisitions were evaluated by experienced operators (EAA and ZD) for proficiency in appropriate acquisition, ensuring avoidance of foreshortening. Following the educational sessions, a preliminary trial took place during which the skills of each student were reassessed. Operators who did not demonstrate adequate proficiency in proper echocardiographic video clip acquisition were excluded from participating in the study. Nine trainees were included in the training, one participant failed the screening (did not reach the expected proficiency), leaving eight operators.

### 2.2. The AI-Based Tool

**Figure 1:**
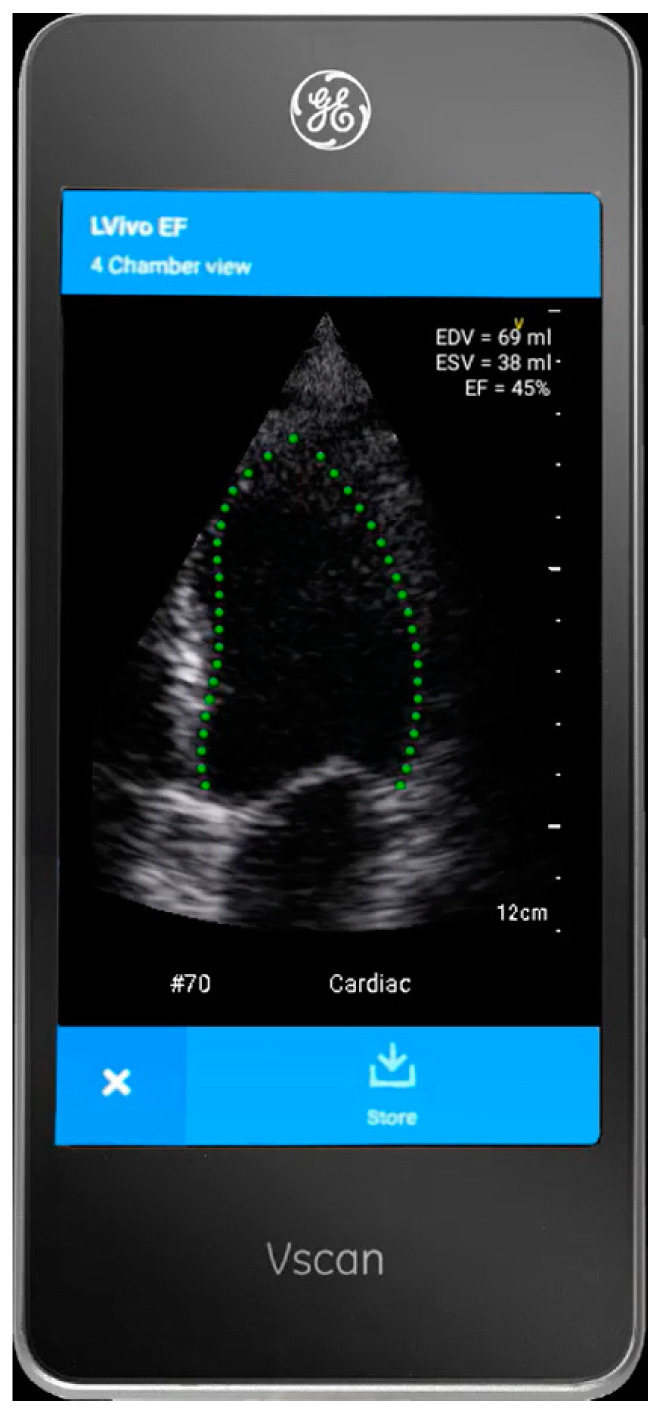
LVivo EF, I AI tool for automatic LVEF evaluation from the A4ch view using the Vscan Extend HUD. Abbreviations: A4ch, apical 4-chamber; AI, artificial intelligence; HUD, hand-held ultrasound device; LVEF, left ventricular ejection fraction.

The HUD (Vscan Extend with Dual Probe; General Electric, Boston, MA, USA) is outfitted with LVivo EF (DiA Imaging Analysis Ltd., Be’er Sheva, Israel; Figure 1), a tool that utilizes AI for automatic calculation of LVEF from the A4ch view. The tool provides immediate real-time measurements of various parameters of the LV, including LVEF, stroke volume, and LV end-diastolic and systolic volume.

### 2.3. Study Protocol

After providing written informed consent, patients underwent the medical student-operated cardiac ultrasound focusing on the A4ch view with optimization of the LV view, avoiding foreshortening. The students were asked to confirm that the interventricular septum is aligned with the vertical plane of the clip and to adjust the depth so that the LV fitted ~70% of the image acquired. Once the view plane and depth were optimally adjusted, the quality was optimized, and the clips were recorded with a minimum of a 2-beat cardiac cycle. The recorded clips were then evaluated by the AI-based tool for LVEF measurement. Reacquisition of the clips was performed in cases of failure of the AI-based software to measure the LVEF, if the automated tracing annotations were not aligned with the endocardial border of the LV, or in case of significant foreshortening of the view (up to five acquisitions were allowed). Patients were excluded if they declined informed consent or if the LVEF assessment by the AI algorithm failed.

Collected data included age, sex, body mass index (BMI), previous medical history, and the course of the admission (including vital signs, laboratory results, and unfavorable outcomes). For the troponin test, we used the high-sensitivity cardiac troponin I (Hs-cTnI) with a rule-out myocardial infarction zone of 6 ng/L and below and a rule-in zone of 65 ng/L and higher. Following admission, patients were assessed for 1-year mortality and cardiovascular-related readmissions based on electronic medical records and remote telephone follow-up.

The main admission diagnoses were divided into acute coronary syndrome (ACS), heart failure, arrhythmias, peri-myocarditis, and others, including admissions for congenital cardiac anomalies, pulmonary embolism, non-coronary procedures (e.g., transcatheter edge-to-edge repair, transcatheter aortic valve implantation, etc.), or other elective-based workups.

### 2.4. Sample Size Calculation

Sample size calculations were formulated to address the objectives of the study. Due to the lack of previous studies exploring this study’s aims, assumptions were necessary to estimate statistical power.

We planned a paired study with a 3:2 control: study ratio, assuming a 1-year event-free survival of 5% vs. 25% between patients with AI-based preserved and reduced LVEF. Based on these assumptions, we calculated that data accrued from 70 participants would suffice to reject the null hypothesis with a probability (power) of 0.8.

Calculations were performed using The PS Power and Sample Size Calculations version 3.1.6 (Vanderbilt University School of Medicine, Nashville, TN, USA), and the type I error was calculated as 0.05 and was two-tailed.

### 2.5. Statistical Analyses

The cohort was divided into participants with preserved (LVEF ≥ 50%) and reduced LV systolic function and statistical analyses were calculated comparing these two subgroups. All characteristics are presented as N (%) for dichotomous variables, mean (± standard deviation) for normally distributed continuous variables, and median (25th and 75th percentiles) for non-normally distributed variables. Differences in baseline and test characteristics are calculated using Chi-square or Fisher’s exact tests for categorical variables, and the t-test or Mann–Whitney U test for continuous variables, where appropriate. Test selection was based on data distribution and normalcy.

The Kaplan–Meier method was used to plot the unadjusted event-free survival curves of the primary endpoint at 1 year comparing patients with AI-based preserved vs. reduced LVEF with the use of Log rank test statistics. Censored cases were graphically presented, when documented. A multivariable Cox’s Proportional Hazards Model was then constructed to compare adjusted event rates between the two cohorts and calculate the Hazard Ratio (HR) with 95% confidence interval (CI). The Cox model was adjusted for age as well as all other pertinent covariates (with *p* < 0.05).

Odds ratio (OR) and CI were then calculated to test the univariate associations between AI-based reduced LVEF and the in-hospital secondary endpoints.

All tests were two-tailed, and a *p*-value of 0.05 or less was considered statistically significant.

Statistical analyses were performed using SPSS Statistics for Windows version 26 (SPSS Inc., Chicago, IL, USA).

## 3. Results

A total of 88 patients were initially recruited for the study. However, measurements were unsuccessful for 6 patients, resulting in a cohort of 82 patients included in the analysis with a mean age of 58.5 ± 16.8 years and 72 (87.8%) male participants, with 34 (41.5%) of them diagnosed with AI-based reduced LVEF. The HUD-based echocardiogram study was conducted within <24 h for 54 (65.9%) patients.

### 3.1. Baseline Demographic and Clinical Characteristics: AI-Based Preserved vs. Reduced LVEF (Table 1)

Patients with AI-based preserved LVEF, as compared to those with LVEF < 50%, had lower rates of known congestive heart failure (CHF) (10.4% vs. 29.4%, *p* = 0.028), and differing rates of main admission diagnoses (*p* = 0.040), i.e., higher rates of acute coronary syndromes (ACS; 60.4% vs. 55.9%), perimyocarditis cases (14.6% vs. 2.9%) and others (10.4% vs. 2.9) and lower rates of heart failure cases (4.2% vs. 20.6%) and arrhythmias (10.4% vs. 16.7%). Patients with preserved LV systolic function vs. reduced function also had higher mean LVEF (57.3 ± 5.3% vs. 38.5 ± 8.1%, *p* < 0.001), higher lowest systolic blood pressure (105.5 ± 12.1 mmHg vs. 98.4 ± 17.1 mmHg, *p* = 0.033) and lower rates of peak Hs-cTnI > 10,000 ng/L (4.2% vs. 38.2%, *p* < 0.001).

### 3.2. Primary Endpoint: Adjusted Association between AI-Based Reduced LV Systolic Function (LVEF < 50%) and Composite Outcome of 1-Year Mortality and Cardiovascular-Related Readmissions

**Figure 2:**
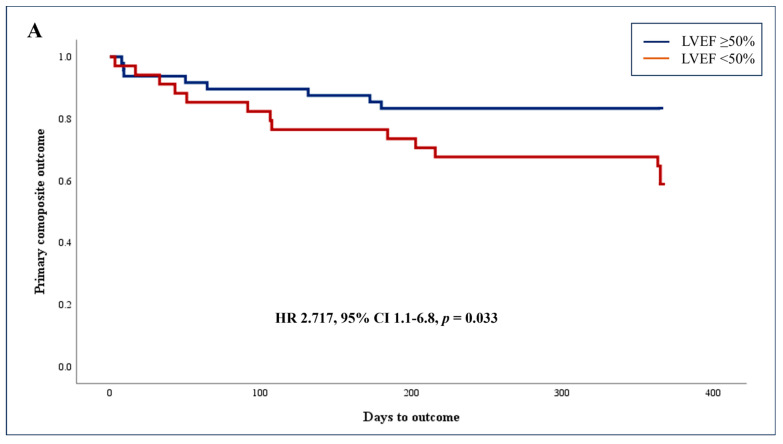

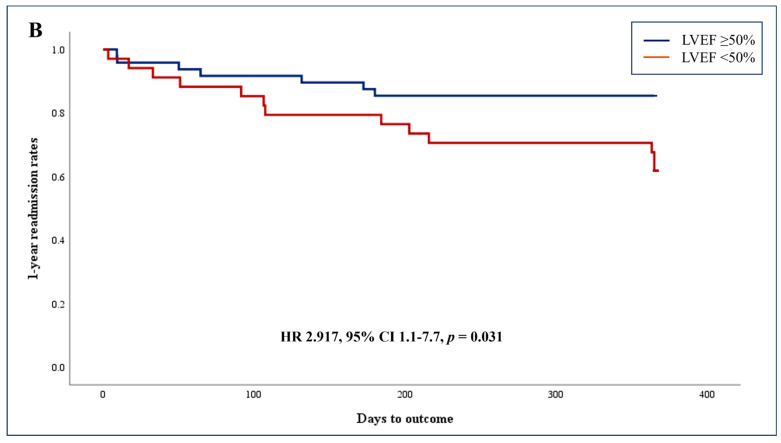
Kaplan–Meier curves of cumulative incidence of the outcomes at 1 year and the multivariable Cox’s Proportional Hazards Model constructed to compare adjusted event rates between the two cohorts calculating the Hazard Ratio with 95% confidence interval. The model was adjusted for age as well as all other pertinent covariates (with *p* < 0.05). (**A**). The 1-year mortality or rehospitalization due to cardiovascular-related causes. (**B**). The 1-year rehospitalization due to cardiovascular-related causes.

Data were available for the entire cohort with no censored cases. The composite outcome, including 1-year mortality and readmissions for cardiovascular causes, was documented in a total of 22 (26.8%) patients, including 2 cases of in-hospital mortality and 20 cases of cardiovascular-related readmissions. The mortality cases comprised of a male patient with AI-based preserved LVEF presenting with leukemia-related pulmonary embolism and a female with AI-based reduced LVEF and an urgent sternotomy for valve replacement with surgery-related complications. Figure 2 presents the Kaplan–Meier curves of the cumulative incidence of the primary composite outcome at 1 year. The unadjusted rates of the composite outcome were higher among patients with AI-based reduced vs. preserved systolic function (41.2% vs. 16.7%, *p* = 0.020; Figure 2A). Similar results were shown when analyzing the rate of 1-year cardiovascular-related readmissions (38.2% vs. 14.6%, *p* = 0.019; Figure 2B).

As detailed in Table 2, multivariable Cox’s Proportional Hazards Model was constructed adjusting for age, ischemic heart disease (IHD), CHF, previous revascularization, and hypertension, revealing that AI-based reduced LVEF predicted the primary composite outcome (HR 2.717, 95% CI 1.083–6.817, *p* = 0.033).

### 3.3. Unadjusted Association of AI-Based Reduced LVEF and Secondary Endpoints

As shown in Table 3, AI-based reduced LVEF was associated with the secondary composite outcome (HR = 4.624, 95% CI 1.619–13.203), shock (HR = 12.185, 95% CI 1.422–104.407), ADHF (HR = 7.077, 95% CI 1.397–35.846) and longer LOS (median [IQR] of 4.7 [2.8, 7.5] vs. 3.0 [1.7, 6.5], *p* = 0.019) as compared with preserved LVEF.

## 4. Discussion

This study showed that the results of an easy-to-use and accessible AI-based program for the automated evaluation of LVEF in the hands of non-experts (in this case medical students) can predict the composite outcome of 1-year mortality and cardiovascular-related readmissions. The unadjusted rates of the composite outcome were higher among patients with AI-based reduced vs. preserved systolic function (41.2% vs. 16.7%) and using the multivariable Cox’s Proportional Hazards Model we revealed that an LVEF < 50% predicted the primary composite outcome. Patients with AI-based reduced LVEF were found to have higher rates of congestive heart failure and arrhythmias and lower rates of ACS and peri-myocarditis. Reduced LVEF also had an unadjusted association with multiple in-hospital secondary unfavorable outcomes.

In alignment with much of the existing literature in the field, our previous article tested the feasibility and accuracy of this AI-based tool, focusing on the clinical validation of the given algorithms [31]. This initial validation step is important to verify the absence of biases in the datasets. There is the concept of “domain shift” where the derivation dataset does not match the real-world dataset leading to underperformance of the algorithms [32]. In the present research, we moved one step forward, studying the ability of the AI tool to predict clinical outcomes.

Using the Cox multivariable model and after adjusting for pertinent variables, we found that only reduced AI-based LVEF, previous revascularization, and hypertension predicted the primary composite outcome (mainly comprised of the 1-year cardiovascular-related readmission component [91%]). Surprisingly, history of IHD or CHF was not associated with the composite outcome. The likely explanation of these results is that the 1-year readmission was driven by patients initially hospitalized with ACS characterized by complex coronary anatomy, higher rates of uncontrolled comorbidities (including smoking), or late presentation, all potentially leading to higher rates of reduced LVEF. As ACS comprised ~59% of the diagnoses of the index admission, the composite outcome mainly stemmed from the high-risk IHD patients and was less affected by the low rates of patients with baseline known CHF admitted for heart failure exacerbation.

In terms of AI and imaging, there are many examples of validation studies. In the field of musculoskeletal imaging, the use of AI can improve fracture detection by emergency medicine clinicians [33]. Similarly, AI/deep learning technology improved residents’ ability to detect hip fractures to the level of fellowship-trained attendings without the use of the program [34]. However, there are some studies in the field of musculoskeletal radiology that use AI as a predictor. One study showed that in those with tibial shaft fracture it could be used to predict the probability of occult posterior malleolar fracture [35]. Another AI application was designed to help identify those at risk for infection after an operation for tibial shaft fractures [36]. In the field of lung imaging, it has been shown to be accurate in identifying the presence or absence of lung sliding (one of the main ultrasonographic signs for pneumothorax) [37], pneumonia in pediatric patients [38], COVID-19 pneumonia [39], and fluid overload in dialysis patients [40].

For cardiac patients, AI is being used in combination with electrocardiogram (ECG) findings to aid in diagnosis. For example, it was utilized to diagnose severely reduced LVEF (≤ 35%) but was not able to identify a specific ejection fraction [41]. Attia and colleagues showed that an AI algorithm in combination with a one-lead ECG-enabled digital stethoscope was able to accurately identify patients with an LVEF ≤ 40% [42]. The combination of AI and ECG has also been used to predict hypertrophic cardiomyopathy, particularly among younger patients [43]. Another important application is the ability to predict atrial fibrillation with its known complications of stroke, in patients with sinus rhythm [44,45]. AI was also shown to diagnose AF when combined with Holter electrocardiogram monitoring data. Taniguchi et al. revealed that when based on a convolutional neural network (CNN) algorithm, AI shows an accuracy of 95.3% (95% CI: 0.952–0.955) for AF detection with the area under the receiver operating characteristic curve (AUROC) of 0.988 (95% CI: 0.987–0.988) [46]. A preliminary study also suggested that an AI-based Holter recording analysis could be significantly accurate for the detection of cardiac arrhythmias, with a shortened analysis duration as compared to a classical approach [47]. Interestingly, Lu and colleagues performed a unique study and used AI to identify atrial fibrillation on echocardiography with an initial AUROC of 0.95 [48].

In terms of AI and cardiac imaging, a large randomized controlled study showed that the evaluation of LVEF by AI was non-inferior to that of cardiac sonographers. In addition, the AI program saved time and the cardiologist could not distinguish between the assessments by the sonographers versus those by AI [49]. Similarly, a study of 28 first-year critical care fellows using AI-assisted measurement of velocity time integral (VTI) increased the accuracy of calculating cardiac output. However, one of the limitations of this study is that all of the trainees performed the VTI measurements on a single healthy patient. This leaves open the question as to how the algorithm would perform on patients of a different body habitus or varied pathologies [50].

Perhaps more importantly, AI can be used to detect rare congenital heart diseases. Arnaout et al. developed AI algorithms to distinguish normal from abnormal hearts in fetal screening ultrasound with a 100% negative predictive value [51]. Other areas of research in the field of AI for transthoracic echocardiography include identifying pulmonary hypertension [52], atrial septal defects in children [53], and rheumatic heart disease [54].

AI is also being developed for transesophageal echocardiography (TEE). Steffner et al. showed that AI could accurately be used to classify TEE views [55]. Yu and colleagues developed an algorithm for TEE to automatically calculate the mitral annular plane systolic excursion (MAPSE) which allows the evaluation of LV function. Using Bland–Altman analysis they found that autoMAPSE (AI generated MAPSE) compared to manual measurement had low bias (0.4 mm) and acceptable limits of agreement (−3.7 to 4.5 mm) [56].

While our original study looked at the ability of medical student non-experts plus AI to accurately evaluate LVEF, other research is looking at AI in resource-limited settings. For example, in Lesotho, nurses and nursing assistants were trained to obtain the parasternal long axis view on a HUD. A total of 83.3% could be evaluated by AI which reinforces a major lesson from our original study with medical students that the main determinant for non-expert operators is image acquisition [57].

It has been shown that the LVEF is a very significant predictor of unfavorable outcomes among patients when performed by experienced operators. Solomen et al. showed that the hazard ratio for all-cause mortality increased by 39% for every 10% reduction in LVEF < 45% among 7599 patients with known heart failure [58]. Similarly, Curtis et al. showed among 7788 stable heart failure patients that higher LVEF measurements were associated with a linear decrease in mortality up to an LVEF of 45% [59]. The importance of LVEF as a predictor was also shown in a broad range of patients. Angaran et al. showed that quantitative echocardiographic LVEF stratified the risk of death and hospitalization among 27,323 patients in a wide range of clinical settings, including during noncardiac admissions when dividing LVEF into the following four categories: <25%, 25–35%, and 36–45% [60]. Unlike previous studies, the novelty of our current manuscript is that the echocardiograms were acquired by novice operators.

## 5. Limitations

This prospective study was carried out at a single center and may be subject to the relevant biases. We also attempted to reach high-performance standards for the operators with a preliminary test, thorough a didactic course, and concluding exam prior to participation, standards that are not necessarily shared by different novice operators. This may lead to participant characteristics and quality of the focused echocardiograms to differ between different centers and operators. As such, the generalizability of our report may be limited. Also, even though it appropriately addressed the research design and sample size calculation, the limited cohort size may expose our analyses to confounders. Furthermore, we did not immediately accept the automated assessment of the LVEF by the AI-based tool and tried to optimize the accuracy of the AI-based calculations. We confirmed that the acquisitions by the medical students were exactly in accordance with the prerequisites as per our methodology and allowed up to 5 acquisition attempts confirming that the automated tracing annotations aligned well with the endocardial border. It is likely that these high standards had a significant role in the study’s results and it is possible that without applying these measures, the AI-based results were less accurate and less clinically relevant. There were cases where the AI -based automated tracings clearly did not properly track the endocardial borders requiring either repeated acquisition or exclusion of the study.

## 6. Conclusions

POCUS is increasingly employed by non-expert medical personnel across various disciplines, contributing to diagnostic processes and decision-making, a trend that was further expanded by the incorporation of multiple cheaper and user-friendly miniature ultrasound devices. The present study showed that when integrated with medical student-operated focused echocardiography using a HUD, AI-based LV systolic function assessment is a potentially powerful clinical tool independently predicting the composite outcome endpoint of 1-year death and cardiovascular-related readmission. AI-based reduced LVEF was also associated with several unfavorable in-hospital outcomes, including the secondary composite outcome, shock, ADHF, and longer LOS. Hand-held focused ultrasound utilization for AI-based assessment of the LV systolic function may thus be used as an important tool for risk stratification and prognostication of patients hospitalized in a cardiology department when incorporated by non-expert personnel, potentially including physicians, nurses and physician’s assistance. These finding should be confirmed in a larger prospective study adjusting for a larger number of pertinent confounders.

## Figures and Tables

**Table 1:** Demographics and clinical and admission characteristics of participants with AI-based preserved LVEF vs. reduced LVEF.

Variable	All Cohort *n* = 82	Preserved LVEF *n* = 48	Reduced LVEF *n* = 34	*p*-Value
**Baseline characteristics**				
Age, mean ± SD	58.5 ± 16.8	56.8 ± 17.1	61.0 ± 16.3	0.266
Male, *n* (%)	72 (87.8)	43 (89.6)	29 (85.3)	0.734
Body mass index, mean ± SD	28.2 ± 4.5	28.5 ± 4.2	27.8 ± 4.9	0.492
**Clinical characteristics**				
Smoking, *n* (%)	28 (34.1)	15 (31.3)	13 (38.2)	0.637
Diabetes mellitus, *n* (%)	22 (26.8)	11 (22.9)	11 (32.4)	0.342
Hypertension, *n* (%)	37 (45.1)	22 (45.8)	15 (44.1)	0.878
Hyperlipidemia, *n*(%)	41 (50.0)	24 (50.0)	17 (50.0)	1.000
Ischemic heart disease, *n* (%)	29 (35.4)	16 (33.3)	13 (38.2)	0.647
Revascularization, *n* (%)	18 (22.0)	9 (18.8)	9 (26.5)	0.342
CKD, *n* (%)	11 (13.4)	6 (12.5)	5 (14.7)	0.773
Atrial fibrillation, *n* (%)	8 (9.8)	5 (10.4)	3 (8.8)	1.000
CHF, *n* (%)	15 (18.3)	5 (10.4)	10 (29.4)	**0.028**
Hypothyroidism, *n* (%)	7 (8.5)	5 (10.4)	2 (5.9)	0.694
**Chronic medications**				
Anti-platelets, *n* (%)	35 (42.7)	22 (45.8)	13 (38.2)	0.493
Anticoagulation, *n* (%)	6 (7.3)	3 (6.3)	3 (8.8)	0.688
Diuretics, *n* (%)	13 (15.9)	9 (18.8)	4 (11.8)	0.543
β-blockers, *n* (%)	26 (31.7)	15 (31.3)	11 (32.4)	0.916
ACE-I/ARB, *n* (%)	20 (24.4)	13 (27.1)	7 (20.6)	0.500
Anti-diabetic drugs, *n* (%)	21 (25.6)	11 (22.9)	10 (29.4)	0.507
Statins, n (%)	33 (40.2)	21 (43.8)	12 (35.3)	0.442
**Hospitalization’s main diagnosis**				**0.040**
Acute coronary syndrome, *n* (%)	48 (58.5)	29 (60.4)	19 (55.9)	
Heart failure, *n* (%)	9 (11.0)	2 (4.2)	7 (20.6)	
Arrhythmias, *n* (%)	11 (13.4)	5 (10.4)	6 (16.7)	
Perimyocarditis, *n* (%)	8 (9.8)	7 (14.6)	1 (2.9)	
Others, *n* (%)	6 (7.3)	5 (10.4)	1 (2.9)	
**In-hospital vitals and laboratory workup**				
AI-based LVEF, mean ± SD	49.5 ± 11.4	57.3 ± 5.3	38.5 ± 8.1	**<0.001**
HR (admission; bpm), mean ± SD	73.9 ± 13.3	72.1 ± 14.2	76.4 ± 11.4	0.144
SBP (admission; mmHg), mean ± SD	141.0 ± 28.7	144.2 ± 30.6	136.6 ± 25.8	0.248
DBP (admission; mmHg), mean ± SD	82.3 ± 16.7	82.3 ± 17.1	82.2 ± 16.5	0.964
O_2_ saturation (admission; %), mean ± SD	95.2 ± 3.1	95.5 ± 2.9	94.8 ± 3.4	0.333
Lowest SBP (mmHg), mean ± SD	102.5 ± 14.8	105.5 ± 12.1	98.4 ± 17.1	**0.033**
Creatinine (admission; mg/dL), mean ± SD	1.0 ± 0.3	1.0 ± 0.4	1.0 ± 0.2	0.809
Peak Hs-cTnI > 10,000 ng/L, n (%)	15 (18.3)	2 (4.2)	13 (38.2)	**<0.001**
Potassium (admission; mEq/L), mean ± SD	3.9 ± 0.4	3.9 ± 0.4	4.0 ± 0.4	0.847
Sodium (admission; mEq/L), mean ± SD	138.6 ± 2.5	138.8 ± 2.3	138.4 ± 2.8	0.591
LDL (admission; mg/dL), mean ± SD	111.0 ± 41.0	111.6 ± 42.7	110.3 ± 39.8	0.905
Hemoglobin (admission; g/dL), mean ± SD	13.8 ± 2.1	13.6 ± 2.0	14.1 ± 2.2	0.267
TSH (mIU/L), mean ± SD	1.9 ± 1.5	1.9 ± 1.5	2.0 ± 1.5	0.670
CRP (peak; mg/L), median [IQR]	0.5 [0.2, 2.3]	0.5 [0.2,2.3]	0.3 [0.2, 2.0]	0.847

Abbreviations. ACE-I, angiotensin-converting enzyme; AI, artificial intelligence; ARB, angiotensin II receptor blocker; bpm, beats per minute; CHF, congestive heart failure; CKD, chronic kidney disease; CRP, C-reactive protein; DBP, diastolic blood pressure; dL, deciliter; g, gram; HR, heart rate; Hs-cTnI, high-sensitivity cardiac troponin I; IQR, interquartile range; L, liter; LVEF, left ventricular ejection fraction; mg, milligram; mIU, milli-international units; mmHg, millimeter of mercury; *n*, number; SD, standard deviation; SBP, systolic blood pressure.

**Table 2:** A multivariable Cox’s Proportional Hazards Model constructed to compare adjusted event rates of 1-year rates of death or cardiovascular-related readmission between the AI-based reduced left ventricular ejection fraction (LVEF) as well as other pertinent covariates (including age and covariates with *p* < 0.05) calculating the Hazard Ratio (HR) with 95% confidence interval (CI).

Variable	Unadjusted Analysis			Adjusted Anslysis		
	HR	95% CI	*p*-Value	HR	95% CI	*p*-Value
Age	1.008	0.982–1.034	0.566	0.977	0.947–1.007	0.133
Reduced LVEF	2.700	1.132–6.441	**0.025**	2.717	1.083–6.817	**0.033**
Congestive heart failure	2.555	1.039–6.286	**0.041**	1.375	0.497–3.806	0.540
Ischemic heart disease	2.526	1.091–5.852	**0.031**	0.494	0.129–1.890	0.303
Hypertension	2.885	1.175–7.082	**0.021**	3.183	1.018–9.957	**0.047**
Previous revascularization	6.074	2.606–14.155	**<0.001**	7.555	1.849–30.867	**0.005**

**Table 3:** The association between AI-based reduced LVEF and serious adverse events.

Variable	All Cohort *n* = 82	AI-Based Preserved LVEF *n* = 48	AI-Based Reduced LVEF *n* = 34	*p*-Value	Unadjusted OR (95% CI)
Secondary composite outcome *, *n* (%)	22 (26.8)	7 (14.6)	15 (44.1)	**0.004**	4.624 (1.619–13.203)
In-hospital death, *n* (%)	2 (2.4)	1 (2.1)	1 (2.9)	1.000	1.424 (0.086–23.593)
Advanced ventilatory support, *n* (%)	8 (9.8)	3 (6.3)	5 (14.7)	0.266	2.586 (0.574–11.655)
Shock, *n* (%)	8 (9.8)	1 (2.1)	7 (20.6)	**0.008**	12.185 (1.422–104.407)
ADHF, *n* (%)	20 (24.4)	6 (12.6)	14 (41.2)	**0.003**	7.077 (1.397–35.846)
LV/LAA thrombus, *n* (%)	6 (7.3)	1 (2.1)	5 (14.7)	0.077	8.103 (0.901–72.867)
Acute kidney injury, *n* (%)	10 (12.2)	3 (6.3)	7 (20.6)	0.084	3.889 (0.927–16.319)
Any ventilatory support, *n* (%)	25 (30.5)	11 (22.9)	14 (41.2)	0.077	2.355 (0.903–6.143)
Cardioversion, *n* (%)	5 (6.1)	1 (2.1)	4 (11.8)	0.155	6.267 (0.668–58.786)
CIED insertion, *n* (%)	3 (3.7)	3 (6.3)	0 (0.0)	0.260	
LOS (days), median [IQR]	3.6 [2.0, 7.0]	3.0 [1.7, 6.5]	4.7 [2.8, 7.5]	**0.019**	
LOS > 3.6 days, *n* (%)	42 (51.2)	20 (41.7)	22 (64.7)	**0.042**	2.567 (1.035–6.362)

* Composite outcome included: in-hospital death, advanced ventilatory support (high-flow nasal cannula, non-invasive positive airway pressure support, and invasive ventilation), shock and acute decompensated heart failure. ADHF, acute decompensated heart failure; AI, artificial intelligence; CI, confidence interval; CIED, cardiovascular implantable electronic device; IQR, interquartile range; LAA, left atrial appendage; LOS, length of stay; LV, left ventricular; LVEF, LV ejection fraction; *n*, number; OR, odds ratio.

## Data Availability

The data presented in this study are available on request from the corresponding author.
